# Supplementary material for: Selective activator of human ClpP triggers cell cycle arrest to inhibit lung squamous cell carcinoma
Source: Nat Commun. 2023 Nov 3;14:7069. doi: 10.1038/s41467-023-42784-4 (PMC10624687; doi:10.1038/s41467-023-42784-4)
Supplement: Supplementary file 1 — Supplementary information [file 41467_2023_42784_MOESM1_ESM.pdf]

Supplementary Information

Selective activator of human ClpP triggers cell cycle arrest to inhibit lung squamous cell carcinoma

Lin-Lin Zhou[1,2,#], Tao Zhang[1,#], Yun Xue[3,4,#], Chuan Yue[5,1], Yihui Pan[2,6], Pengyu Wang[6], Teng Yang[1,6], Meixia Li[7], Hu Zhou[2,6,8], Kan Ding[2,7], Jianhua Gan[9], Hongbin Ji[3,4,10*], Cai-Guang Yang[1,2,5,6*]

1. State Key Laboratory of Drug Research, Centre for Chemical Biology, Shanghai Institute of Materia Medica, Chinese Academy of Sciences, Shanghai 201203, China.
2. University of Chinese Academy of Sciences, Beijing 100049, China.
3. State Key Laboratory of Cell Biology, Shanghai Institute of Biochemistry and Cell Biology, Center for Excellence in Molecular Cell Science, Chinese Academy of Sciences, Shanghai 200031, China
4. School of Life Science, Hangzhou Institute for Advanced Study, University of Chinese Academy of Sciences, Hangzhou 310024, China.
5. School of Chinese Materia Medica, Nanjing University of Chinese Medicine, Nanjing 210023, China.
6. School of Pharmaceutical Science and Technology, Hangzhou Institute for Advanced Study, University of Chinese Academy of Sciences, Hangzhou 310024, China.
7. Carbohydrate-Based Drug Research Center, CAS Key Laboratory of Receptor Research, State Key Laboratory of Drug Research, Shanghai Institute of Materia Medica, Chinese Academy of Sciences, Shanghai 201203, China
8. Analytical Research Center for Organic and Biological Molecules, State Key Laboratory of Drug Research, Shanghai Institute of Materia Media, Chinese Academy of Sciences, Shanghai 201203, China
9. School of Life Sciences, Fudan University, Shanghai 200433, China.
10. School of Life Science and Technology, Shanghai Tech University, Shanghai 200120, China.

#. These authors contributed equally to this work.

*. Correspondence, hbji@sibcb.ac.cn (H.J.), yangcg@simm.ac.cn (C.-G.Y.)

Supplementary Table 1. Data collection and refinement statistics.

	ZK53/*Hs*ClpP (8HGK)
**Data collection** [a]	
Space group	P 1 21 1
Cell dimensions	
a, *b*, *c* (Å)	120.3, 97.0, 123.8
α, β, γ (°)	90.0, 94.0, 90.0
Resolution (Å) [b]	20.53-1.90 (1.95-1.90)
No. of observations	1527775
No. unique	223016
R_{merge} [c]	0.110 (1.702)
$I/\sigma(I)$	10.4 (1.2)
Completeness (%)	99.9 (100.0)
Redundancy	6.9 (7.1)
**Data refinement**	
Resolution (Å)	20.53-1.90
No. reflections	212014
R_{work}/R_{free}	0.186/0.221
No. atoms	
protein	18972
ligand	364
water	478
Average B value (Å^2)	
protein	33.4
ligand	78.1
water	46.0
RMSD [d] in	
Bond lengths (Å)	0.009
Bond angles (°)	1.329
Ramachandran	
Preferred (%)	98.1
Allowed (%)	1.9

[a] The structure was resolved using a single crystal.

[b] Highest resolution shell is shown in parenthesis.

[c] $R_{merge} = \Sigma|(I - \langle I \rangle)|/\Sigma(I)$, where I is the observed intensity.

[d] Root mean squared deviation.

Supplementary Table 2. The residues with disordered electron density in the X-ray structure of ZK53/*Hs*ClpPΔ56 complex.

	Omitted residues[a]	Residues with truncated side chain[b]
Chain A	181-192, 250-277	R71, K112, Q218, M233
Chain B	57, 65-69, 181-192, 250-277	R71, K112, E197, K203, K211, K214, E237
Chain C	57, 66-69, 181-192, 250-277	K112, E197, K214, Q218, M233, E237
Chain D	57, 66-68, 181-192, 250-277	E70, R71, K112, K113, E197, Q218, E237
Chain E	57, 181-192, 250-277	E64, R68, R71, R81, K112, E237
Chain F	57, 65-69, 181-192, 250-277	R71, K112, E237
Chain G	57, 65-68, 181-192, 250-277	R71, K112, K203, K211, K214, Q218, M233
Chain H	57, 64-70, 181-192, 250-277	R71, R81, K112, E197, E237
Chain I	57, 68-69, 182-192, 250-277	E64, Q65, E68, R71, R81, K113, K203, Q218, E237
Chain J	64-69, 181-191, 250-277	E70, R71, E109, K112, K203, Q218, E237
Chain K	57, 66-68, 181-192, 250-277	R71, R81, K112, K203, E237
Chain L	57, 66-67, 181-192, 250-277	R68, K112, K113, K203
Chain M	57, 181-192, 250-277	E64, R68, R81, K112, Q179, K214, Q218, E221, E237
Chain N	57, 181-192, 250-277	E64, Q65, R68, E70, R71, K112, E197, M233, E234, E237

[a] The residues were omitted from the model due to poorly ordered electron density.

[b] The side chains of the residues exhibiting low electron density were truncated.

Supplementary Table 3. Complete blood count analysis of Balb/c mice (n = 4) treated with ZK53.

Test name (units)	Vehicle control (mean ± SD)	80 mg/kg ZK53 (mean ± SD)
WBC (10^9/L)	3.35 ± 0.70	1.35 ± 0.47
LYM# (10^9/L)	1.57 ± 0.58	0.48 ± 0.21
MON# (10^9/L)	0.63 ± 0.18	0.40 ± 0.11
GRA# (10^9/L)	1.15 ± 0.25	0.47 ± 0.16
LYM (%)	45.02 ± 9.87	34.18 ± 5.92
MON (%)	18.9 ± 3.68	30.72 ± 2.85
GRA (%)	36.08 ± 11.43	35.10 ± 4.40
HGB (g/L)	146.62 ± 7.78	143.82 ± 5.24
MCH (pg)	35.88 ± 7.13	39.35 ± 0.54
MCHC (g/L)	699.5 ± 124.71	757.75 ± 23.22
RBC (10^{12}/L)	4.24 ± 0.88	3.65 ± 0.16
MCV (fL)	51.4 ± 4.25	52.12 ± 1.05
HCT (%)	21.52 ± 3.43	19 ± 1.17
RDWSD (fL)	33.25 ± 6.02	35.75 ± 1.09
RDWCV (%)	15.98 ± 1.84	17.1 ± 0.62
PLT	664.5 ± 401.06	381.75 ± 26.49
PCT (%)	0.92 ± 0.33	0.67 ± 0.04
MPV (fL)	15.78 ± 3.22	17.58 ± 0.49
PDW (fL)	25.875 ± 3.48	27.35 ± 2.02
P-LCR (%)	62.22 ± 21.77	76.98 ± 3.52

Supplementary Table 4. Plasma biochemical analysis of Balb/c mice (n = 4) treated with ZK53.

Test name (units)	Vehicle control (mean ± SD)	80 mg/kg ZK53 (mean ± SD)
ALT (U/L)	310.75 ± 23.32	241.5 ± 25.5
AST (U/L)	3.75 ± 1.09	4.75 ± 1.48
ALP (U/L)	73 ± 7.28	92 ± 10.2
ALB (g/L)	42.75 ± 0.43	47.25 ± 1.09
TP (g/L)	64.5 ± 1.8	65 ± 1
TB (μmol/L)	9.52 ± 1.14	4.76 ± 0.79
GLU (mmol/L)	5.75 ± 0.94	4.86 ± 0.42
UREA (mmol/L)	3.22 ± 0.043	3.48 ± 0.6
CR (μmol/L)	84.5 ± 15.37	59.75 ± 9.28
TC (μmol/L)	1.35 ± 0.013	1.32 ± 0.173
TG (μmol/L)	0.44 ± 0.017	0.44 ± 0.038
IGG (mg/mL)	34.61 ± 4.24	36.65 ± 3.34
CK (U/mL)	0.26 ± 0.037	0.27 ± 0.046
A/G	1.98 ± 0.19	2.67 ± 0.162

Supplementary Table 5. Comparisons of *Hs*ClpP activators.

	ZK53	D9	ADEP 4	ONC201	ONC212
EC_{50} (μM)	0.22	0.22	0.9	0.5	0.85
substrate	FITC-casein	FITC-casein	α-casein	FITC-casein	α-casein
Selectivity	High selectivity on human ClpP	High selectivity on human ClpP	No selectivity	Selectivity for some bacterial ClpP	No selectivity
Flexibility	Simple and flexible scaffold	Simple but rigid scaffold	Rigid macrocyclic scaffold	Rigid tricyclic scaffold	Rigid tricyclic scaffold
Anticancer effects	Inhibited LUSC in vitro and in mouse models	N/A	Cytotoxicity in HEK293 T-REx cells	Inhibited acute myeloid leukemia cell proliferation and tumor growth in mouse models	

N/A, not available

Supplementary Table 6. Information about the bacteria strains used in this work.

Strain	Description	Source
Newman	Wild-type, *S. aureus* ATCC 25904	Lab stock
LR	*Limosilactobacillus reuteri* BNCC 186563	Lab stock
LGG	*Lactobacillus rhamnosus GG* ATCC53103	Lab stock
BL	*Bifidobacterium longum subsp. Infantis* BNCC 185971	Lab stock
DH5α	*E. coli* plasmid cloning host	Lab stock
BL21 (DE3) Gold	*E. coli* plasmid expression host	Lab stock
Rosetta (DE3)	*E. coli* plasmid expression host	Lab stock

Supplementary Table 7. Primer sequences used in this study.

Primer	Sequence (5’-3')
CLPP-F	CCATCTACGACACGATGCAG
CLPP-R	CATGATCTCCTCTGCCTGGA
GAPDH-F	GCCGCATCTTCTTTTGCGTC
GAPDH-R	TGAAGGGGTCATTGATGGCA
CCNA2-F	CGCTGGCGGTACTGAAGTC
CCNA2-R	GAGGAACGGTGACATGCTCAT
CCNE2-F	TCAAGACGAAGTAGCCGTTTAC
CCNE2-R	TGACATCCTGGGTAGTTTTCCTC
PCNA-F	CCTGCTGGGATATTAGCTCCA
PCNA-R	CAGCGGTAGGTGTCGAAGC
CDK2-F	CCAGGAGTTACTTCTATGCCTGA
CDK2-R	TTCATCCAGGGGAGGTACAAC
MT-ND1-F	CCCTAAAACCCGCCACATCT
MT-ND1-R	GAGCGATGGTGAGAGCTAAGGT
HGB-F	GTGCACCTGACTCCTGAGGAGA
HGB-R	CCTTGATACCAACCTGCCCAG

Supplementary Figures

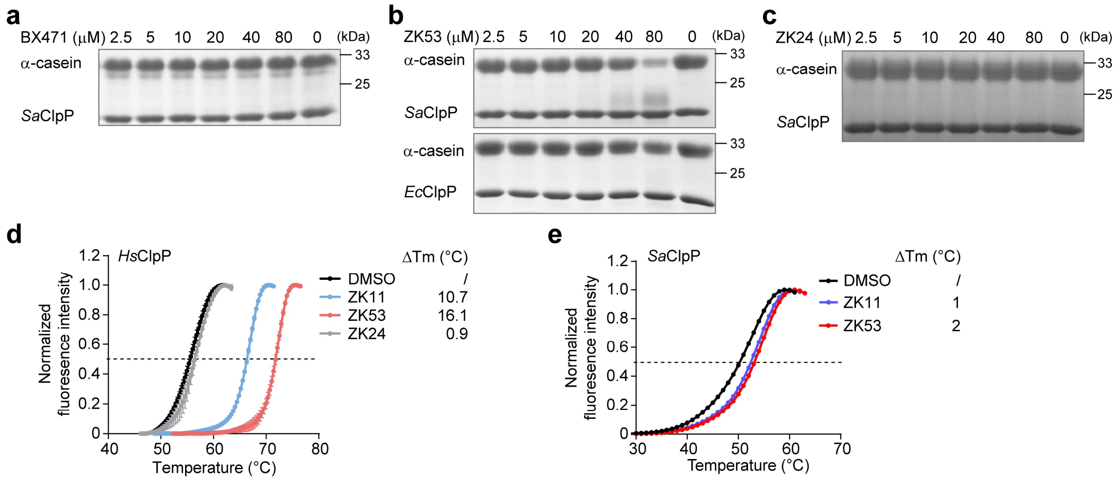

Supplementary Fig. 1 Effects of our activators on α-casein hydrolysis by ClpP and thermal stability of ClpP in vitro. a Effect of BX471 on α-casein hydrolysis by *Sa*ClpP in the PAGE-based assay. Representative gel of three independent experiments is shown. **b** Effect of ZK53 on α-casein hydrolysis by *Sa*ClpP and *Ec*ClpP in the PAGE-based assay. Representative gels of three independent experiments are shown. **c** Effect of ZK24 on α-casein hydrolysis by *Sa*ClpP in the PAGE-based assay. Representative gel of three independent experiments is shown. **d** Effect of our activators on the thermal stability of *Hs*ClpP in the DSF assay (n = 3 biological samples). The shift of melting temperature (ΔTm) of *Hs*ClpP is indicated. **e** Effect of ZK11 and ZK53 on the thermal stability of *Sa*ClpP in the DSF assay (n = 3 biological samples). The ΔTm values of *Sa*ClpP are indicated.

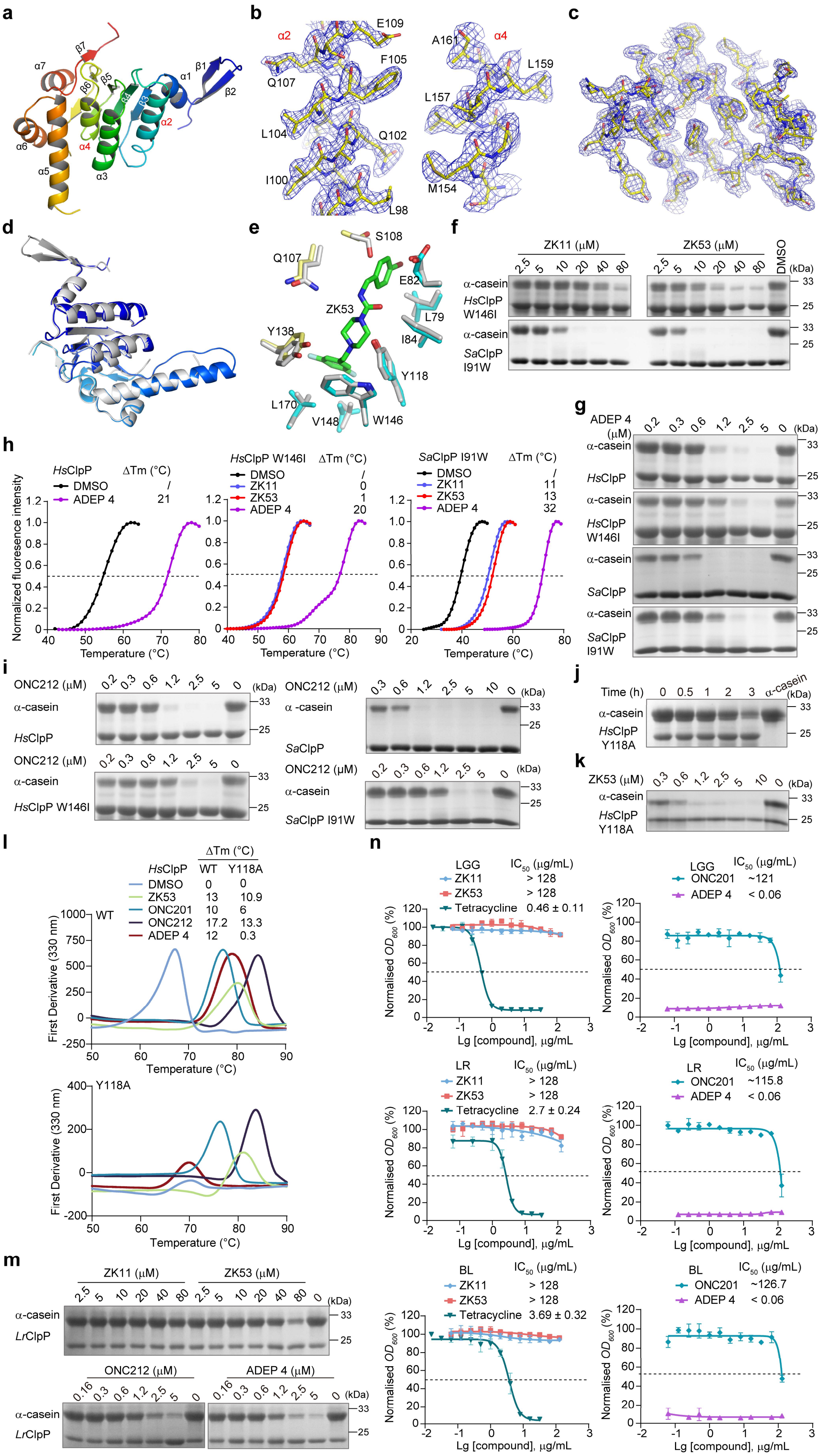
a
α7
β7
β6
β5
β4
β3
α1
β1
β2
α6
α4
α2
α5
α3
b
α2
E109
F105
Q107
L104
Q102
I100
L98
A161
α4
L159
L157
M154
c
d
e
S108
Q107
E82
ZK53
L79
I84
Y138
Y118
L170
V148
W146
f
ZK11 (μM)
ZK53 (μM)
2.5 5 10 20 40 80 2.5 5 10 20 40 80 DMSO (kDa)
α-casein
HsClpP W146I
α-casein
SaClpP I91W
33
25
g
ADEP 4 (μM) 0.2 0.3 0.6 1.2 2.5 5 0 (kDa)
α-casein
HsClpP
HsClpP W146I
SaClpP
SaClpP I91W
h
HsClpP ΔTm (°C)
DMSO /
ADEP 4 21
HsClpP W146I ΔTm (°C)
DMSO /
ZK11 0
ZK53 1
ADEP 4 20
SaClpP I91W ΔTm (°C)
DMSO /
ZK11 11
ZK53 13
ADEP 4 32
Normalized fluoresence intensity
Temperature (°C)
i
ONC212 (μM) 0.2 0.3 0.6 1.2 2.5 5 0 (kDa)
α-casein
HsClpP
HsClpP W146I
ONC212 (μM) 0.3 0.6 1.2 2.5 5 10 0 (kDa)
α -casein
SaClpP
ONC212 (μM) 0.2 0.3 0.6 1.2 2.5 5 0 (kDa)
SaClpP I91W
j
Time (h) 0 0.5 1 2 3 α-casein (kDa)
α-casein
HsClpP Y118A
k
ZK53 (μM) 0.3 0.6 1.2 2.5 5 10 0 (kDa)
α-casein
HsClpP Y118A
l
ΔTm (°C)
HsClpP WT Y118A
DMSO 0 0
ZK53 13 10.9
ONC201 10 6
ONC212 17.2 13.3
ADEP 4 12 0.3
WT
Y118A
First Derivative (330 nm)
Temperature (°C)
m
ZK11 (μM)
ZK53 (μM)
2.5 5 10 20 40 80 2.5 5 10 20 40 80 0 (kDa)
α-casein
LrClpP
ONC212 (μM)
ADEP 4 (μM)
0.16 0.3 0.6 1.2 2.5 5 0 0.16 0.3 0.6 1.2 2.5 5 0 (kDa)
α-casein
LrClpP
n
LGG IC50 (μg/mL)
ZK11 > 128
ZK53 > 128
Tetracycline 0.46 ± 0.11
LGG IC50 (μg/mL)
ONC201 ~121
ADEP 4 < 0.06
LR IC50 (μg/mL)
ZK11 > 128
ZK53 > 128
Tetracycline 2.7 ± 0.24
LR IC50 (μg/mL)
ONC201 ~115.8
ADEP 4 < 0.06
BL IC50 (μg/mL)
ZK11 > 128
ZK53 > 128
Tetracycline 3.69 ± 0.32
BL IC50 (μg/mL)
ONC201 ~126.7
ADEP 4 < 0.06
Normalised OD600 (%)
Lg [compound], μg/mL

Supplementary Fig. 2 Mechanism of selectivity of ZK53 and effects of ZK53 on *Lr*ClpP activation and gut microbes' growth. a Presentation of *Hs*ClpP monomer with α2 and α4 locating at the periphery and core of *Hs*ClpP, respectively. **b** 2fo-fc map contoured to 1.2σ (blue mesh) showing the electron density of α2 and α4 in our *Hs*ClpP structure. The amino acids are presented as yellow sticks. **c** 2fo-fc density map contoured to 1.0σ (blue mesh) of the ligand-binding area in chain A. **d** Superimposition of the monomeric structures of *Hs*ClpP (blue cartoon, PDB: 1TG6) and ZK53/*Hs*ClpP complex (gray cartoon, PDB: 8HGK). **e** Close view of the ligand-binding pocket in **d** showing the effect of ZK53 binding on the conformation of key residues. ZK53 is presented as green stick. **f** Effect of ZK11 and ZK53 on α-casein hydrolysis by W146I *Hs*ClpP and I91W *Sa*ClpP mutants in the PAGE-based assay. The reaction was cultured for 2 h before subjected to PAGE analysis. Representative gels of three independent experiments are shown. **g** Effect of ADEP 4 on α-casein hydrolysis by *Hs*ClpP, W146I *Hs*ClpP, *Sa*ClpP, and I91W *Sa*ClpP in the PAGE-based assay. The reaction was cultured for 2 h before subjected to PAGE analysis. Representative gels of three independent experiments are shown. **h** Effect of ClpP activators on the thermal stability of *Hs*ClpP, W146I *Hs*ClpP, and I91W *Sa*ClpP in the DSF assay (*n* = 3 biological samples). ΔTm values of ClpP proteins are indicated. **i** Effect of ONC212 on α-casein hydrolysis by *Hs*ClpP, W146I *Hs*ClpP, *Sa*ClpP, and I91W *Sa*ClpP in the PAGE-based assay. The reaction was cultured for 2 h before subjected to PAGE analysis. Representative gels of three independent experiments are shown. **j** The α-casein hydrolysis by Y118A ClpP mutant in the PAGE-based assay. Representative gels of three independent experiments are shown. **k** Effect of ZK53 on α-casein hydrolysis by Y118A ClpP in the PAGE-based assay. The reaction was cultured for 2 h before subjected to PAGE analysis. Representative gel of three independent experiments is shown. **l** Effect of ClpP activators on the thermal stability of *Hs*ClpP, and Y118A *Hs*ClpP in the NanoDSF assay. ΔTm values of ClpP proteins are indicated (*n* = 3 biological samples). **m** Effect of ClpP activators on α-casein hydrolysis by *Lr*ClpP in the PAGE-based assay. Representative gels of three independent experiments are shown. **n** Growth curve of gut microbes treated with ClpP activators (*n* = 3 biological samples). Tetracycline was assayed as a positive control. The data are represented as mean ± SD

(error bars).

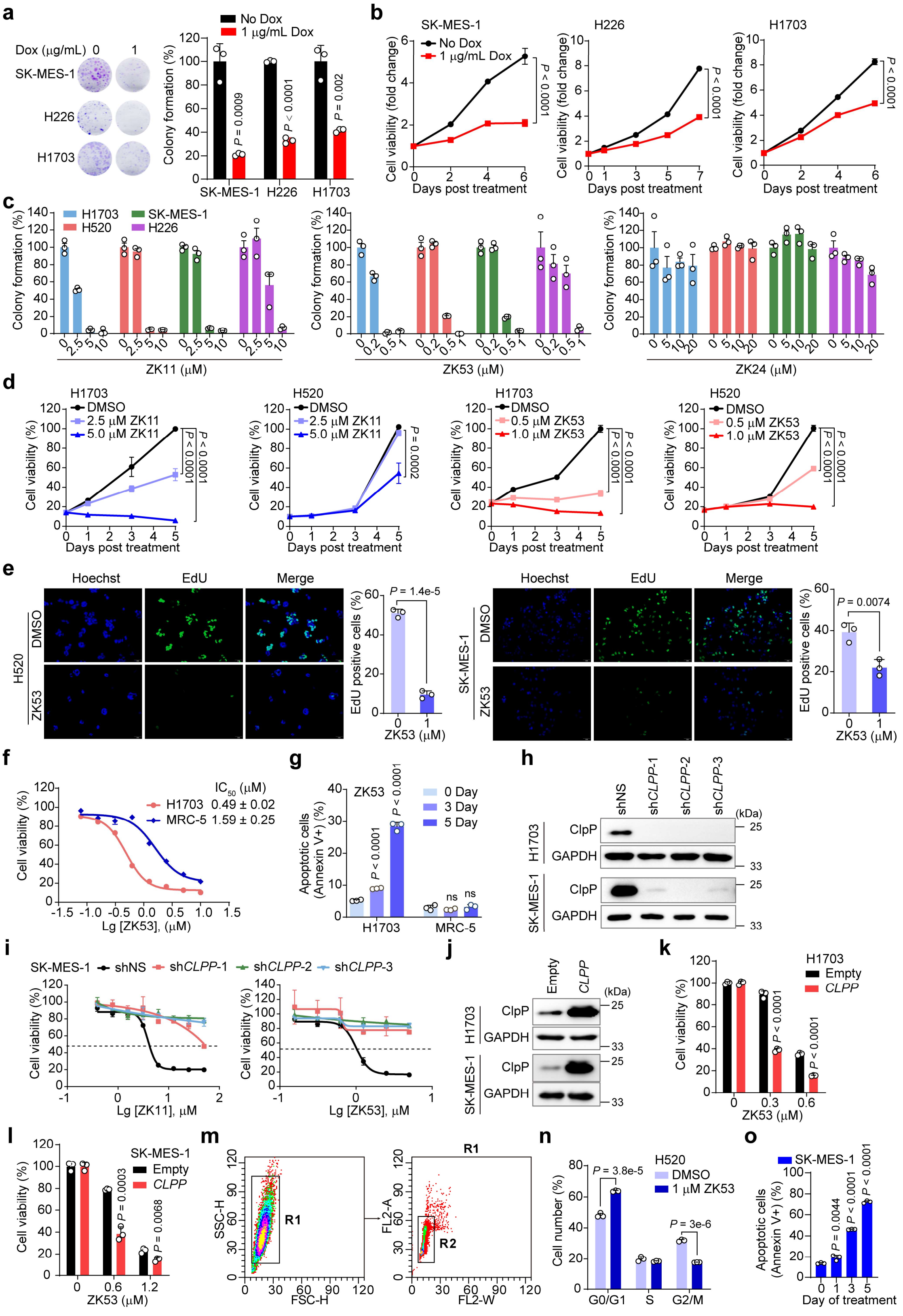

Supplementary Fig. 3 ZK53 inhibits LUSC cell proliferation in a ClpP-dependent manner. a Effect of inducible Y118A ClpP overexpression on colony formation of LUSC

cells in clonogenic assay (*n* = 3 biological samples). The *P* values are calculated by two-sided Student's *t* test with confidence interval of 95%. **b** Effect of Y118A ClpP overexpression on proliferation of SK-MES-1, H226, and H1703 cell lines (*n* = 3 biological samples). The *P* values are calculated by two-sided Student's *t* test with confidence interval of 95%. **c** Quantitative results of clonogenic assay showing the effect of ZK11, ZK53, and ZK24 on colony formation in H1703, H520, SK-MES-1, and H226 cell lines (*n* = 3 biological samples). **d** Effect of ZK11 and ZK53 on cell viability of H1703 and H520 cells in growth curve assay (*n* = 3 biological samples). The *P* values are calculated by one-way ANOVA with Dunnett's multiple comparisons test. **e** Representative images showing the inhibitory effect of ZK53 on H520 and SK-MES-1 cells in the EdU proliferation assay (*n* = 3 biological samples). The percentage of EdU positive (green) cells to total cell count (blue) is calculated. The *P* values were calculated by a two-sided Student's *t*-test with confidence interval of 95%. Scale bar, 50 µm. **f** Inhibitory effect of ZK53 on the cell viability of H1703 and MRC-5 cell lines. The IC_{50} values are shown (*n* = 3 biological samples). **g** Apoptosis analysis of H1703 and MRC-5 cells treated with 1 µM ZK53 for the indicated time points (*n* = 3 biological samples). The *P* values were calculated by two-way ANOVA. **h** The protein level of ClpP in H1703 and SK-MES-1 cell lines with *CLPP* knockdown. The images shown are representative of three independent experiments. **i** Effect of ZK11 and ZK53 on the viability of shNS and sh*CLPP* SK-MES-1 cells (*n* = 3 biological samples). Error bars represent the mean ± SD. **j** The protein level of ClpP in H1703 and SK-MES-1 cells with *CLPP* overexpression. The images shown are representative of three independent experiments. **k, l** Effect of ZK53 on cell viability in H1703 (**k**) and SK-MES-1 (**l**) cells overexpressing empty vector or *CLPP* (*n* = 3 biological samples). Data are represented as mean ± SD (error bars). **m** Gating strategy of cell cycle analysis. The R1 was gated FSC and SSC plots of unstained cell control sample to remove cell debris, R2 was gated to exclude the doublets and cell aggregates by gating in single cells (FL2-1 vs. FL2-W). **n** Quantitation graph of cell cycle analysis of H520 cells upon ZK53-treatment for 48 h (*n* = 3 biological samples). The *P* values are calculated by two-sided Student's *t* test with confidence interval of 95%. **o** Apoptosis analysis of SK-MES-1 cells treated with 1

μM ZK53 for the indicated time points (n = 3 biological samples). The P values were calculated by one-way ANOVA with Dunnett's multiple comparisons test. All data are represented as mean ± SD (error bars).

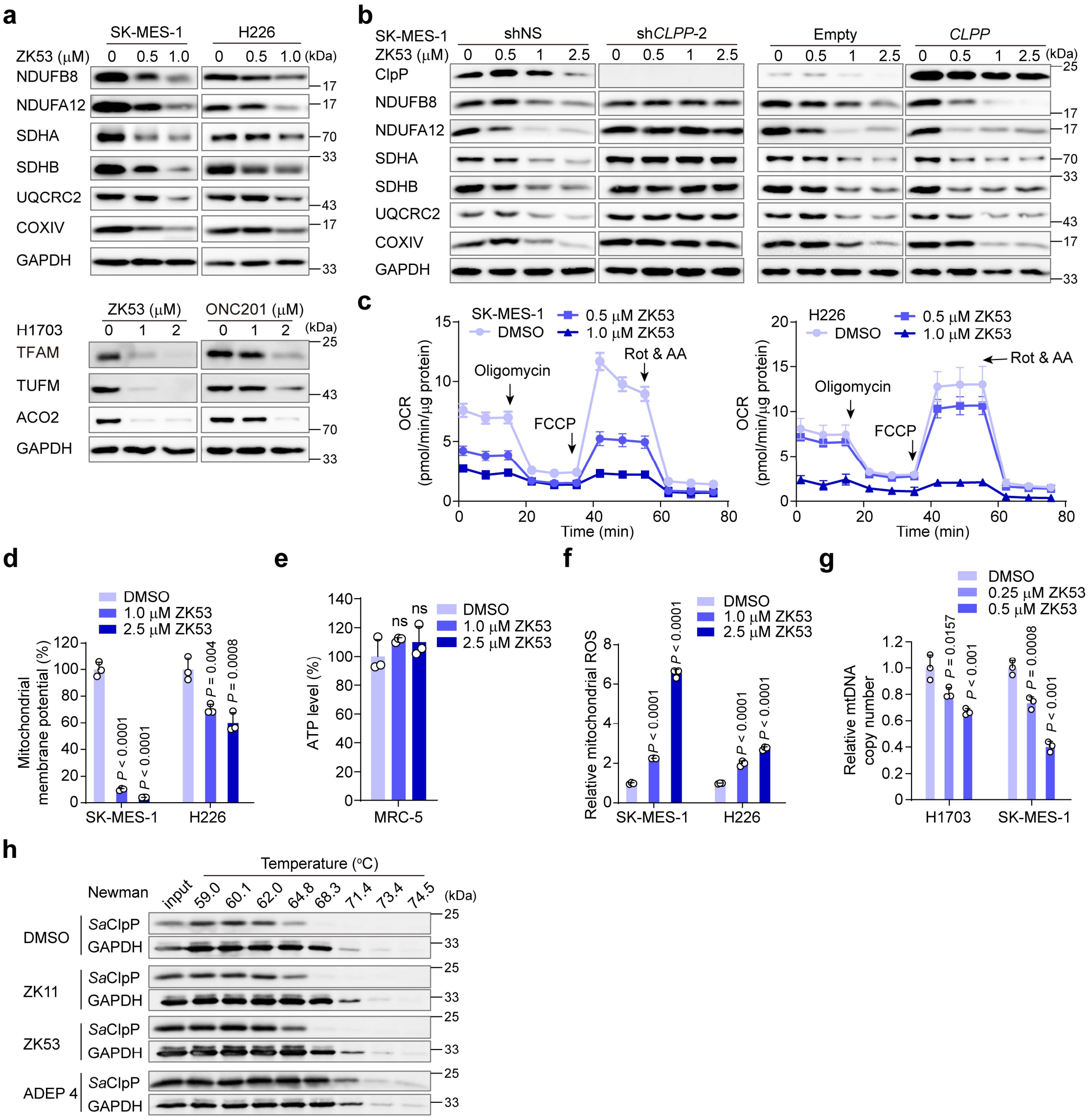

Supplementary Fig. 4 ZK53 impairs mitochondrial proteins and functions in a ClpP-dependent manner in LUSC cells. a Effect of ZK53 on the abundance of mitochondrial proteins in LUSC cells. The images shown are representative of three independent experiments. **b** Immunoblot showing the effect of ZK53 on the protein abundance of ETC subunits in SK-MES-1 cells with *CLPP* knockdown (left two columns) or overexpressing (right two columns). The images shown are representative of three independent experiments. **c** Effect of ZK53 on OCR in SK-MES-1 and H226 cell lines

using Seahorse Analyzer (*n* = 4 biological samples). **d-g** Effect of ZK53 on the mitochondrial membrane potential in SK-MES-1 and H226 cell lines (*n* = 3 biological samples) (**d**), ATP level in H1703 cell lines (*n* = 3 biological samples) (**e**), mitochondrial ROS in SK-MES-1 and H226 cell lines (*n* = 3 biological samples) (**f**), and mtDNA copy number in H1703 and SK-MES-1 cell lines (*n* = 3 biological samples) (**g**). The *P* values are calculated by one-way ANOVA with Dunnett's multiple comparisons test. **h** Effect of ZK11, ZK53 and ADEP 4 on the thermal stability of *Sa*ClpP protein in intact cell of the *S. aureus* Newman strain in CETSA. The gel images shown are representative of three independent experiments. All data are represented as mean ± SD (error bars).

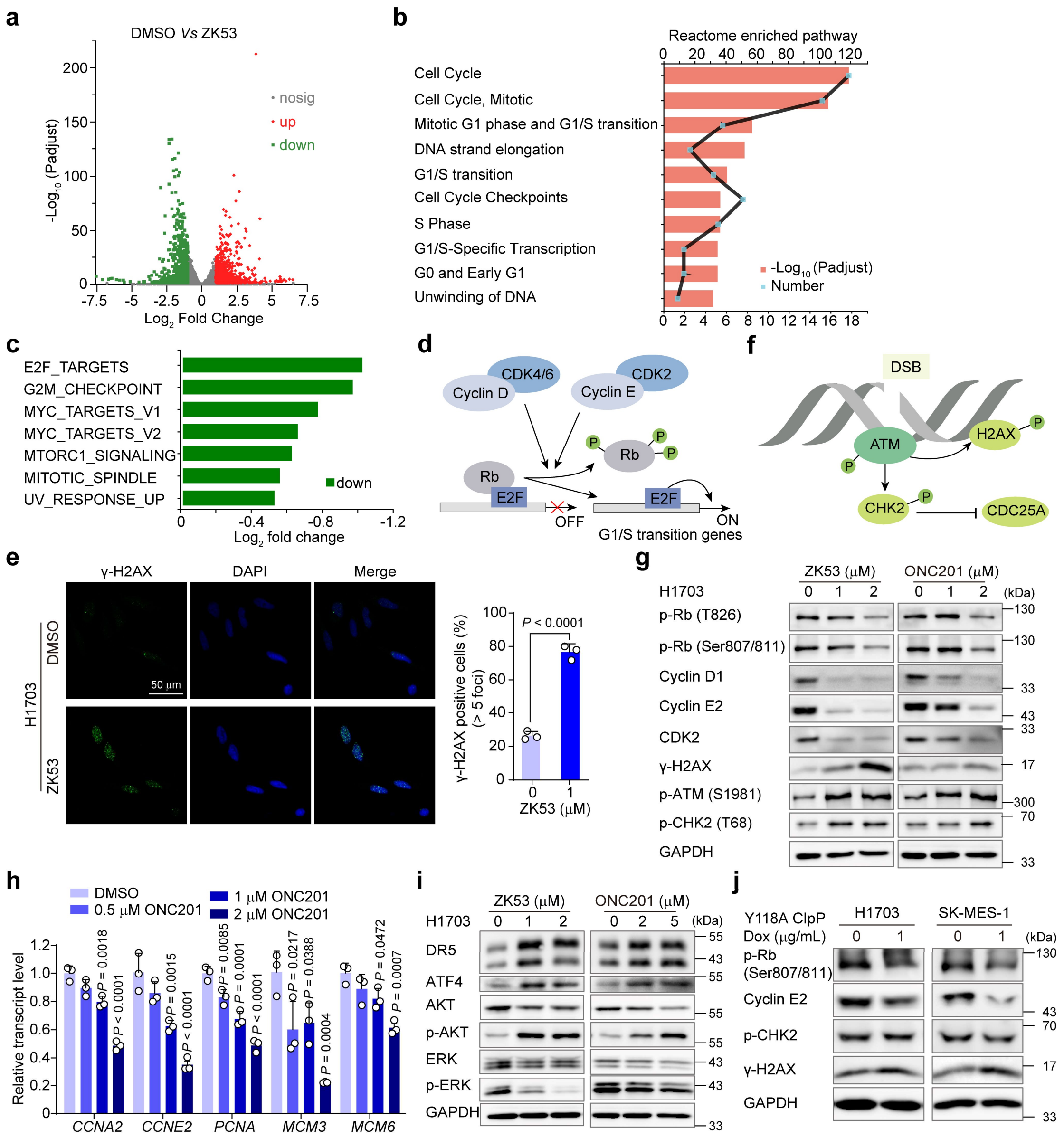

Supplementary Fig. 5 Regulatory pathway and mechanism of ZK53 treatment in H1703 cells. a The volcano plot showing DEGs of the ZK53-treated H1703 cells compared with control. The genes with fold change (|FC|>2) and adjusted *P*-value (*P* adjust <0.05) are selected as DEGs. Each dot represents a gene. **b** Top 10 enriched signaling pathways of the Reactome enrichment analysis in ZK53-treated H1703 cells compared with control. **c** GSVA analysis of DEGs. The down-regulated gene sets are listed. **d** Schematic illustration of Rb-E2F pathway. **e** Effect of ZK53 on γ-H2AX expression in H1703 cells. Representative immunofluorescence (left) and quantitative results (right) of γ-H2AX (green) are shown after H1703 cells were treated with 1 μM ZK53 for 48 h (*n* = 3 biological samples). The cells with more than 5 foci are counted as

positive. Scale bar, 50 µm. The *P* values are calculated by two-sided Student's *t* test with confidence interval of 95%. **f** Schematic illustration of ATM-mediate DNA damage response pathway. **g** Effect of ZK53 and ONC201 on Rb phosphorylation, regulatory proteins involved in the cell cycle, and DDR-related proteins in H1703 cells in immunoblot analysis. The images shown are representative of three independent experiments. **h** Effect of ONC201 on gene expression of E2F targets in H1703 cells measured by qPCR (n = 3 biological samples). The *P* values are calculated by one-way ANOVA with Dunnett's multiple comparisons test. **i** Effect of ZK53 and ONC201 on the reported ONC201-activated pathways in H1703 cells in immunoblot analysis. The images shown are representative of three independent experiments. **j** Effect of Y118A ClpP overexpression on the ZK53-related pathways in H1703 and SK-MES-1 cells in immunoblot analysis. The images shown are representative of three independent experiments.

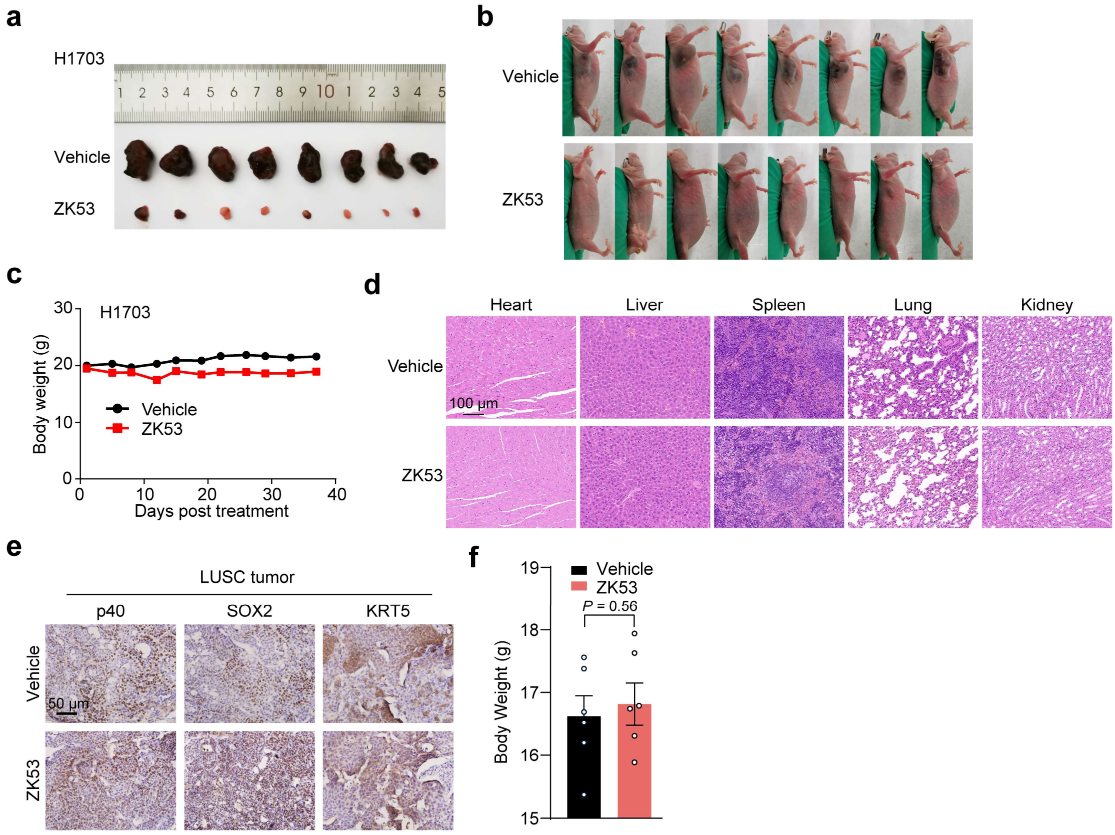

Supplementary Fig. 6 ZK53 shows anticancer effects and low toxicity in xenograft and genetically engineered mouse models. **a** Photograph of the tumors at the endpoint of treatment (*n* = 8 samples). **b** Photograph of the tumor-bearing mice before euthanization (*n* = 8 animals). **c** Body weight of the nude mice treated with ZK53 (*n* = 8 animals). **d** Histological morphology of H&E-stained major organs of mice at the endpoint. Scale bar, 100 μm**.** The images shown are representative of three animals. **e** Representative immunohistochemistry staining for p40, SOX2 and KRT5 in in LUSC tumors of KL mice treated with vehicle or ZK53. Scale bar, 50 μm. The images shown are representative of five animals. **f** Body weights of the KL mice treated with vehicle or ZK53 (*n* = 6 animals). The *P* values were calculated by a two-sided Student's *t*-test. All data are represented as mean ± SEM (error bars).

Supplementary methods

General Information.

All solvents and reagents were purchased from commercial vendors and used without purification. Reactions were monitored using Agilent 1200 (HPLC) and 6110 (MSD) systems with Xbridge C18, 3.5 μm, 4.6 × 50 mm column. HPLC gradient method utilized a 5% to 95% acetonitrile in H_2O with 0.01% trifluoroacetic acid over 5 min with a 1.0 mL/min flow rate. Final products were purified by positive-phase preparatory using a Lisui EZ Plus 100 D with a PDA detector and flash silica gel column. Purification methods used a 30 min gradient from 5% to 50% ethyl acetate in petroleum ether. ^{1}H NMR spectra were recorded on Bruker-400 (400 MHz) and Bruker-600 (600 MHz) spectrometers using D_2O or $CDCl_3$ as solvent. ^{13}C NMR spectra were detected on Bruker-500 (125 MHz) spectrometer using D_2O or $CDCl_3$ as solvent. NMR spectra were processed and analyzed in MestReNova. Low-resolution mass spectra and high-resolution mass spectra (HRMS) were recorded on Agilent 6110 and Agilent G6520 Q-TOF using electrospray positive ionization, respectively. Positive total ion scans were observed from 100 to 1000 m/z. The purity of the final compound was assessed at a wavelength of 254 nm by HPLC analysis, injected with 2 μL sample in MeCN solution. The purities of all target compounds are >95% in HPLC. Compound names were generated using ChemBioDraw Ultra v14.0 systematic naming.

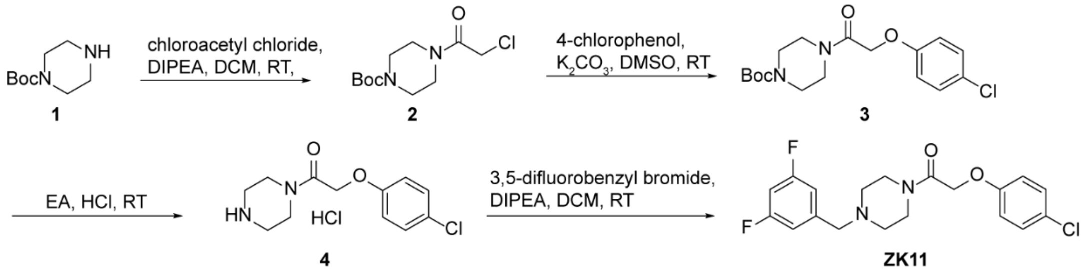

*2-(4-chlorophenoxy)-1-(piperazin-1-yl)ethan-1-one hydrochloride (**4**):* A mixture of **1** (1.86 g, 10 mmol, 1.0 eq), 2,2-diethoxyethanamine (DIPEA) (1.94 g, 15.0 mmol, 1.5 eq) and dichloromethane (DCM) (20 mL) was stirred at room temperature for 5 min.

Chloroacetyl chloride (1.13 g, 10 mmol, 1.0 eq) was dissolved in DCM (10 mL) and added dropwise to the reaction mixture. The reaction was monitored by TLC (hexane/EtOAc (5:1)) and stirred at room temperature for 4 h. The mixture was then washed with saturated $NaHCO_3$. The organic layer was separated, and the aqueous layer was washed with DCM. The combined organic solution was dried over anhydrous Na_2SO_4, the suspension was filtered, and the filtrate was concentrated in vacuo to afford crude compound **2** without further purification. Compound **2**, 4-chlorophenol (1.29 g, 10 mmol, 1.0 eq) and K_2CO_3 (2.07 g, 15 mmol, 1.5 eq) were dissolved in dimethyl sulfoxide (DMSO) and stirred at room temperature overnight, the mixture was washed with water and the organic layer was separated. The combined organic solution was dried over anhydrous Na_2SO_4, the suspension was filtered and the filtrate was concentrated in vacuo to afford compound **3**. Compound **3** was dissolved in 2.0 M hydrochloric acid in ethyl acetate (EA) to 2.0 M and stirred at room temperature for 6 h. The precipitate was filtered and washed with EA to afford compound **4** as a white powder 2.10 g (83 % yield). ^{1}H NMR (400 MHz, D_2O) δ 7.24 (d, *J* = 9.0 Hz, 2H), 6.85 (d, *J* = 9.0 Hz, 2H), 4.81 (m, 4H), 3.74 (s, 4H), 3.24 (d, *J* = 19.6 Hz, 4H). ^{13}C NMR (125 MHz, D_2O) δ 168.7, 155.9, 129.5, 126.2, 116.2, 65.5, 42.8, 41.3, 40.3, 38.6. LC-MS $[M + H]^+$ 255.1.

2-(4-chlorophenoxy)-1-(4-(3,5-difluorobenzyl)piperazin-1-yl)ethan-1-one ***(ZK11)***: Compound **4** (0.39 g, 1.0 mmol, 1.0 eq), 3,5-difluorobenzyl bromide (0.23 g, 1.1 mmol, 1.1 eq), and DIPEA (0.39 g, 3.0 mmol, 3.0 eq) were dissolved in DCM (10 mL) and stirred at room temperature for 6 h. The mixture was washed with water and dried over anhydrous Na_2SO_4. The suspension was filtered and the filtrate was concentrated in vacuo. The crude product was purified by Flash column (PE/EA 10:1 to 2:1) to afford ZK11 as a white powder 0.30 g (78% yield). ^{1}H NMR (400 MHz, $CDCl_3$) δ 7.28 – 7.22 (m, 2H), 6.90 (dt, *J* = 5.7, 3.4 Hz, 4H), 6.78 – 6.66 (m, 1H), 4.69 (s, 2H), 3.74 – 3.63 (m, 2H), 3.63 – 3.55 (m, 2H), 3.49 (s, 2H), 2.54 – 2.35 (m, 4H). ^{13}C NMR (125 MHz, $CDCl_3$) δ 166.1, 164.1, 164.0, 162.1, 162.0, 156.5, 142.0, 129.5, 126.7, 116.0, 111.4, 111.4, 111.3, 111.2, 102.9, 102.7, 102.5, 67.9, 61.8, 53.1, 52.7, 45.3, 42.1. LC-MS $[M + H]^+$ 381.1;

HRMS [M + H]+ (ESI-TOF) calcd for $C_{19}H_{19}ClF_2N_2O_2$ 381.1181, found 381.1179; HPLC purity at 254 nm, 99.2%.

Synthesis of **ZK53**

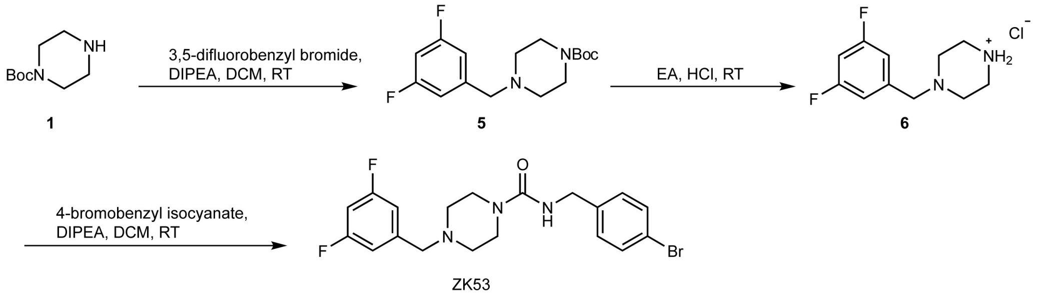

*1-(3,5-difluorobenzyl)piperazine hydrochloride (**6**)*: A mixture of **1** (1.86 g, 10 mmol, 1.0 eq), 3,5-difluorobenzyl bromide (2.07 g, 1.0 mmol, 1.0 eq) and DIPEA (1.94 g, 15.0 mmol, 1.5 eq) in DCM (20 mL) was stirred at room temperature for 6 h. The mixture was washed with water and dried over anhydrous Na_2SO_4, the suspension was filtered, and the filtrate was concentrated in vacuo to afford crude compound **5** without further purification. Compound **5** was dissolved in 2.0 M hydrochloric acid in EA and stirred at room temperature for 6 h. The precipitate was filtered and washed with EA to afford compound **6** as a white powder 1.75 g (70 % yield). ^{1}H NMR (400 MHz, D_2O) δ 7.07 (d, *J* = 5.7 Hz, 2H), 6.99 (m, 1H), 4.39 (s, 2H), 3.53 (s, 9H). ^{13}C NMR (125 MHz, D_2O) δ 164.2, 164.1, 161.7, 161.6, 130.6, 130.5, 114.6, 114.5, 114.4, 114.3, 106.3, 106.1, 105.8, 59.4, 48.1, 40.6. LC-MS $[M + H]^+$ 213.1.

*N-(4-bromobenzyl)-4-(3,5-difluorobenzyl)piperazine-1-carboxamide (**ZK53**)*: Compound **6** (0.25 mg, 1.0 mmol, 1.0 eq) and DIPEA (0.39 g, 3.0 mmol, 3.0 eq) were dissolved in DCM and stirred for 5 min, then 4-bromobenzyl isocyanate (0.34 g, 1.5 mmol, 1.5 eq) was added. The reaction was stirred at room temperature for 6 h. The mixture was washed with water and the organic layer was separated and concentrated in vacuo. The crude product was purified by Flash column (PE/EA 10:1 to 1:1) to afford ZK53 as a white powder 0.36 g (86% yield). ^{1}H NMR (600 MHz, $CDCl_3$) δ 7.44 (d, *J* = 8.3

Hz, 2H), 7.18 (d, J = 8.3 Hz, 2H), 6.87 (m, 2H), 6.70 (m, 1H), 4.76 (t, J = 4.9 Hz, 1H), 4.37 (d, J = 5.6 Hz, 2H), 3.48 (s, 2H), 3.42 – 3.35 (t, J = 3.4 Hz, 4H), 2.46 – 2.38 (t, J = 3.4 Hz, 4H). ^{13}C NMR (125 MHz, $CDCl_3$) δ 164.4, 164.2, 161.9, 161.8, 157.4, 138.6, 131.7, 129.5, 121.1, 111.5, 111.2, 102.6, 62.0, 52.7, 44.3, 43.8. LC-MS $[M + H]^+$ 424.1; HRMS [M + H]+ (ESI-TOF) calcd for $C_{19}H_{20}BrF_2N_3O$ 424.0836, found 424.0832; HPLC purity at 254 nm, 98.9%.

Synthesis of **ZK24**

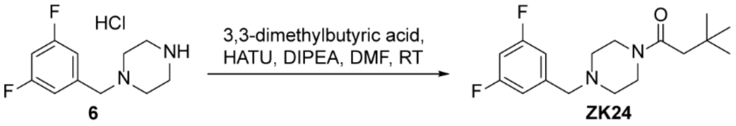

*1-(4-(3,5-difluorobenzyl)piperazin-1-yl)-3,3-dimethylbutan-1-one (**ZK24**)*: 3,3-dimethylbutyric acid (0.12 g, 1.0 mmol, 1.0 eq), 1-Hydroxy-7-azabenzotriazole HOAT (0.16 g, 1.2 mmol, 1.2 eq), 2-(7-aza-1H-benzotriazole-1-yl)-1,1,3,3-tetramethyluronium hexafluorophosphate HATU (0.46 g, 1.2 mmol, 1.2 eq), and DIPEA (.39 g, 3.0 mmol, 3.0 eq) were dissolved in DMF and stirred for 5 min at room temperature. Compound **6** (0.25 mg, 1.0 mmol, 1.0 eq) was added and the mixture was stirred at room temperature for 6 h. The solution was washed with brine and extracted with DCM. The solvent was then removed under vacuo and the mixture was purified by Flash column (PE/EA 10:1 to 2:1) to afford **ZK24** 0.87g (87% yield.). ^{1}H NMR (400 MHz, $CDCl_3$) δ 6.86 (d, J = 6.2 Hz, 2H), 6.68 (m, 1H), 3.71 – 3.59 (m, 2H), 3.56 – 3.48 (m, 2H), 3.47 (s, 2H), 2.44 – 2.36 (m, 4H), 2.24 (s, 2H), 1.03 (s, 9H). ^{13}C NMR (125 MHz, $CDCl_3$) δ 170.4, 164.1, 164.0, 162.1, 162.0, 142.3, 111.4, 111.4, 111.3, 111.2, 102.8, 102.6, 102.4, 61.9, 53.1, 52.9, 46.6, 44.6, 41.3, 31.4, 30.1. LC-MS $[M + H]^+$ 311.2; HRMS [M + H]+ (ESI-TOF) calcd for $C_{17}H_{24}F_2N_2O$ 311.1935, found 311.1931; HPLC purity at 254 nm, 96.6%.

NMR spectrum of ZK11, ZK24 and ZK53

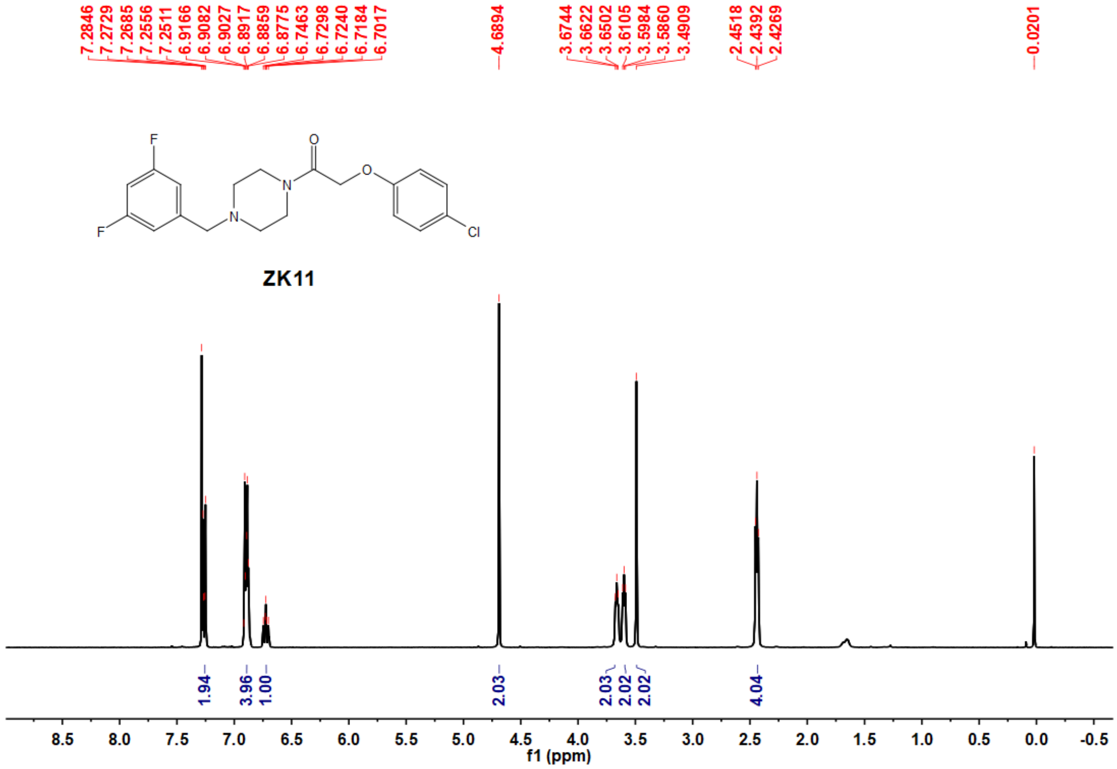

Supplementary Fig. 7 ^{1}H NMR spectrum (400 MHz, $CDCl_3$) of ZK11.

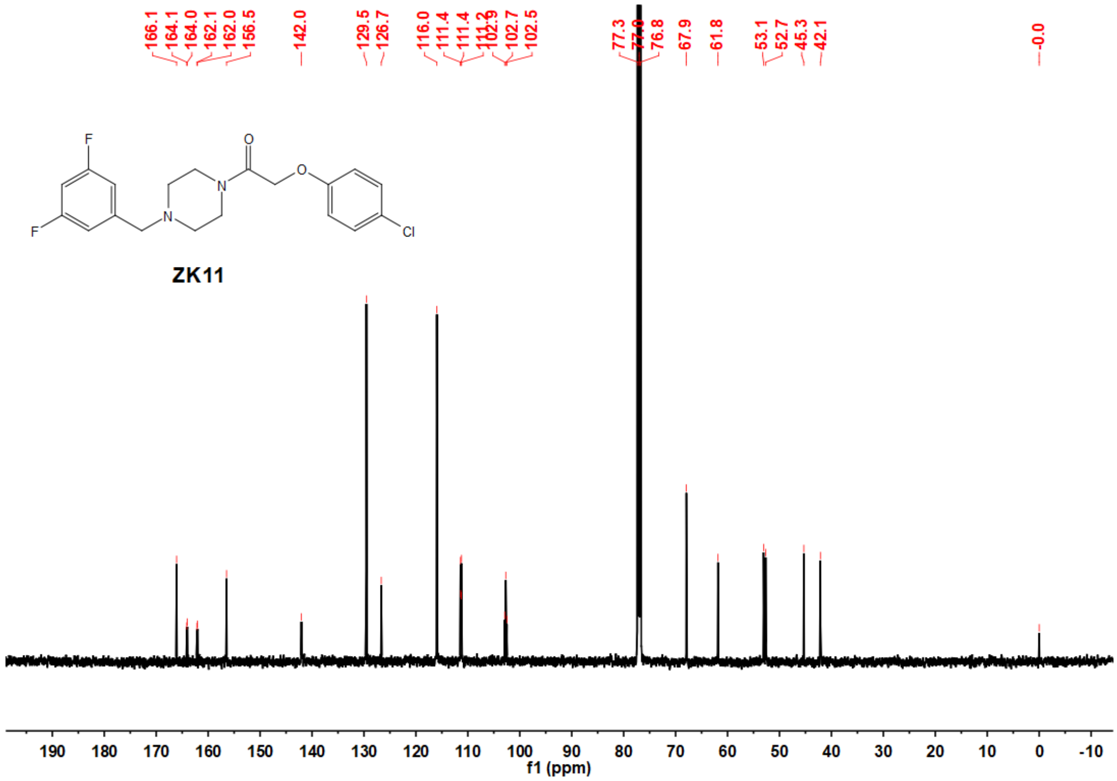

Supplementary Fig. 8 ^{13}C NMR spectrum (125 MHz, $CDCl_3$) of ZK11.

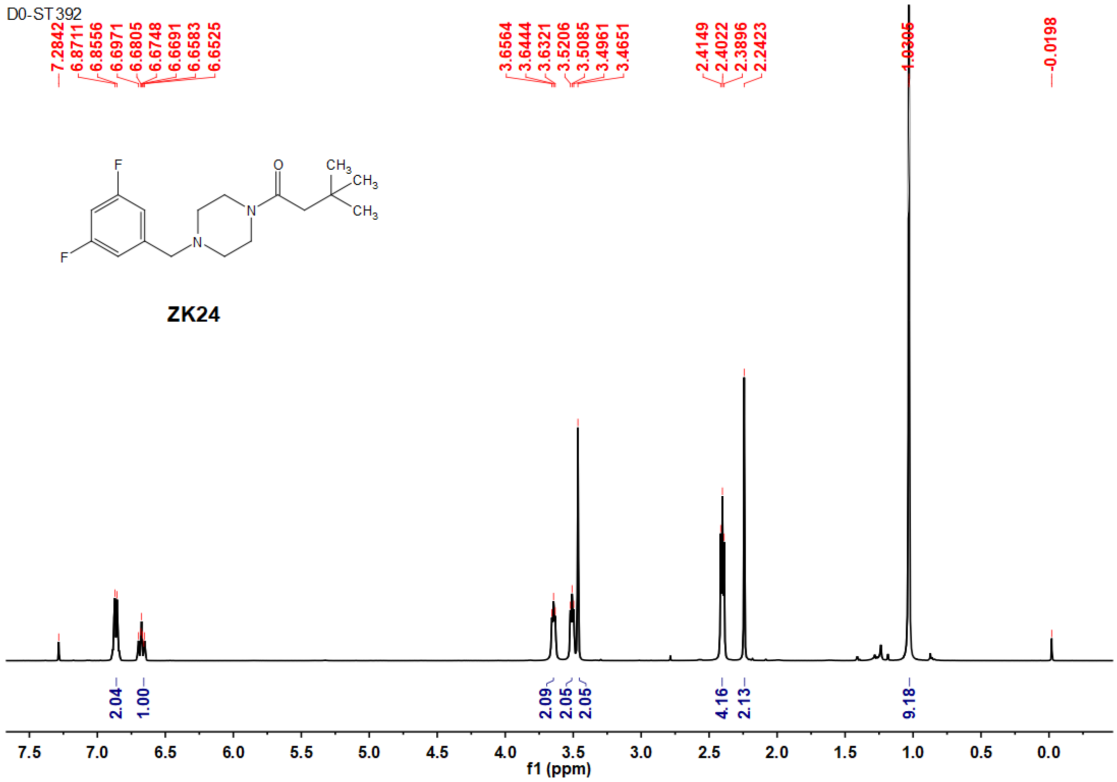

Supplementary Fig. 9 ^{1}H NMR spectrum (400 MHz, $CDCl_3$) of ZK24.

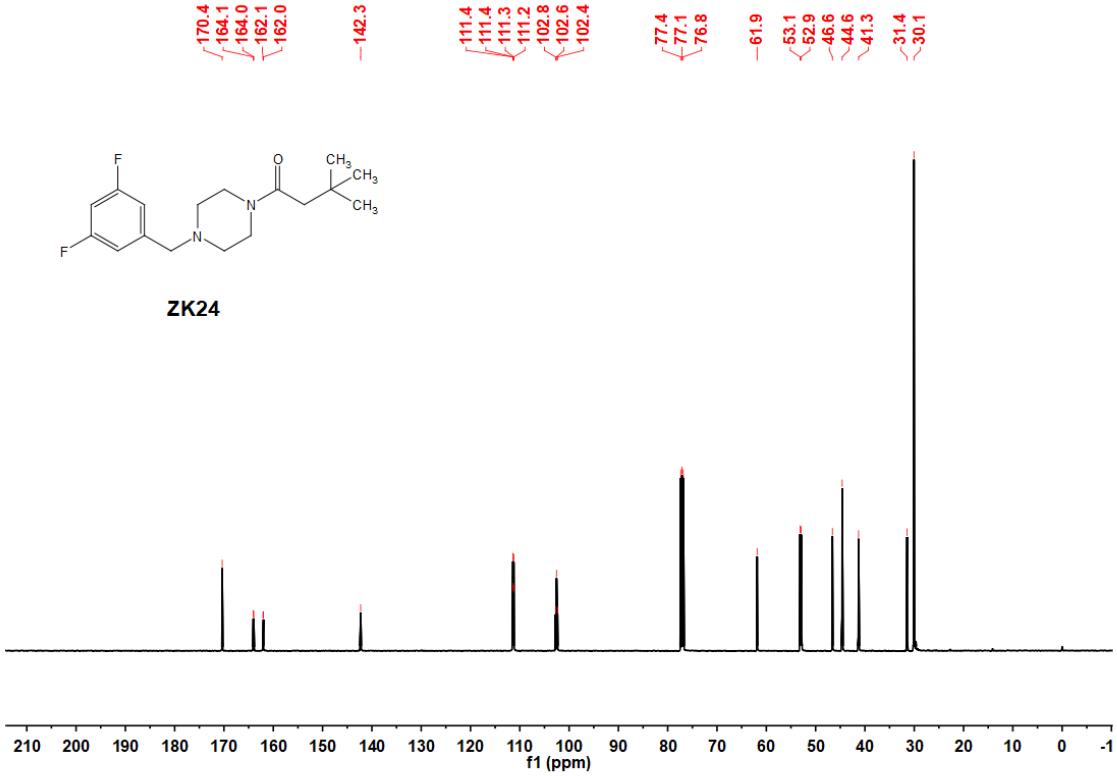

Supplementary Fig. 10 ^{13}C NMR spectrum (125 MHz, $CDCl_3$) of ZK24.

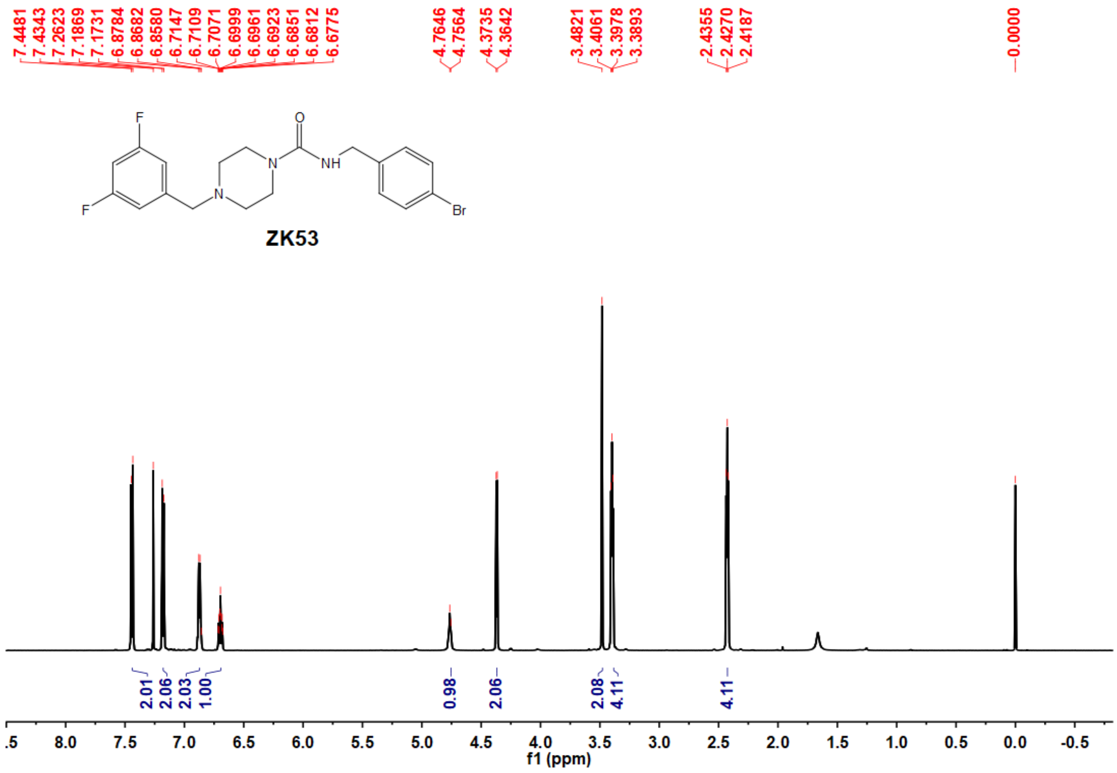

Supplementary Fig. 11 ^{1}H NMR spectrum (600 MHz, $CDCl_3$) of ZK53.

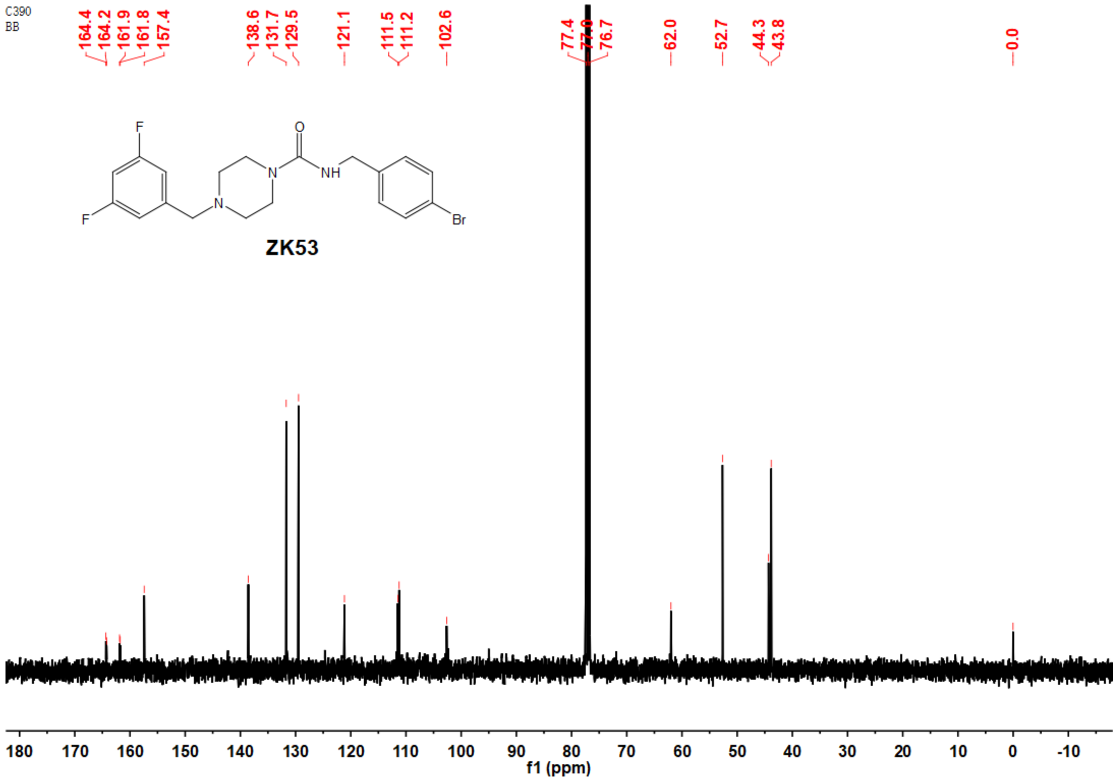

Supplementary Fig. 12 ^{13}C NMR spectrum (125 MHz, $CDCl_3$) of ZK53.

The purities of ZK11, ZK24 and ZK53 analyzed by HPLC

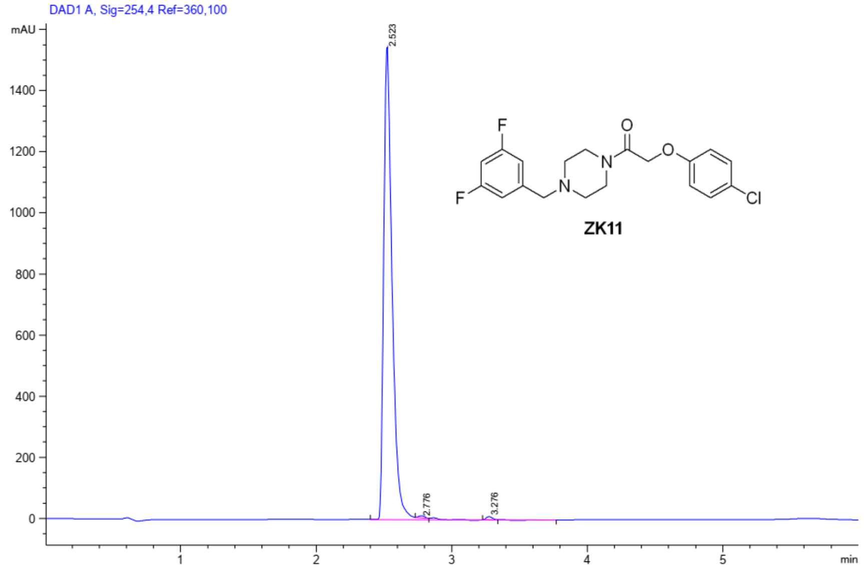

Integration Results for DAD1 A, Sig=254,4 Ref=360,100

RetTim	Width	Area	Height	Area%
2.52	0.07	6673.89	1556.43	99.17
2.78	0.05	23.40	7.03	0.35
3.28	0.05	32.67	10.25	0.49

Supplementary Fig. 13 The purity of ZK11 analyzed by HPLC.

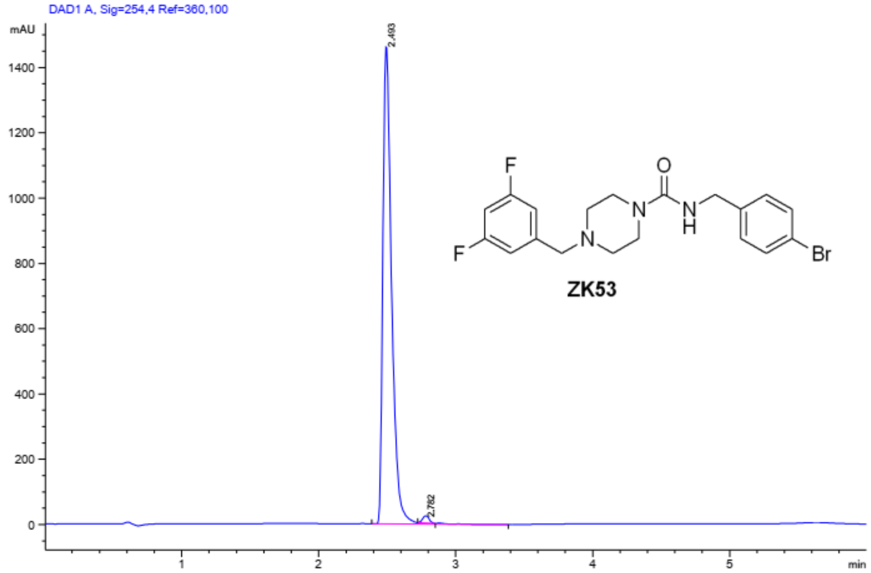

Integration Results for DAD1 A, Sig=254,4 Ref=360,100

RetTim	Width	Area	Height	Area%
2.49	0.07	6487.31	1474.16	98.91
2.78	0.05	71.25	21.99	1.09

Supplementary Fig. 14 The purity of ZK53 analyzed by HPLC.

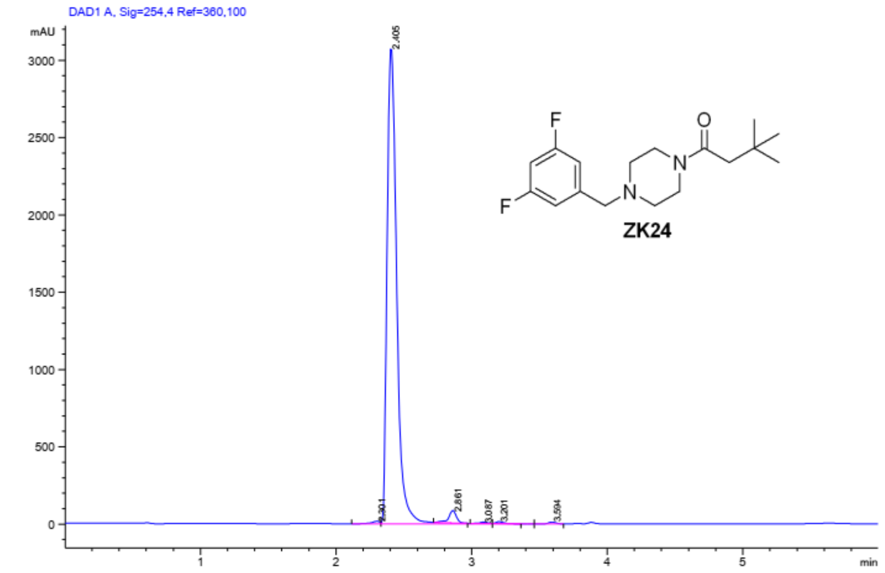

Integration Results for DAD1 A, Sig=254,4 Ref=360,100

RetTim	Width	Area	Height	Area%
2.30	0.06	72.62	16.85	0.46
2.41	0.08	15251.17	3086.73	96.58
2.86	0.06	325.18	81.42	2.06
3.09	0.06	34.90	8.90	0.22
3.20	0.06	55.66	13.09	0.35
3.59	0.06	51.67	12.70	0.33

Supplementary Fig. 15 The purity of ZK24 analyzed by HPLC.

HRMS spectrum of ZK11, ZK24 and ZK53

**Data Filename**	ESIH202204433.d	**Sample Name**	ZK11
**Sample ID**		**Position**	P1-B1
**Instrument Name**	Agilent G6520 Q-TOF	**Acq Method**	20160322_MS_ESIH_POS_1min.m
**Acquired Time**	11/3/2022 17:09:15	**IRM Calibration Status**	Success
**DA Method**	small molecular data analysis method.m	**Comment**	ESIH by fangsu

User Spectra

Fragmentor Voltage	Collision Energy	Ionization Mode
150	0	ESI

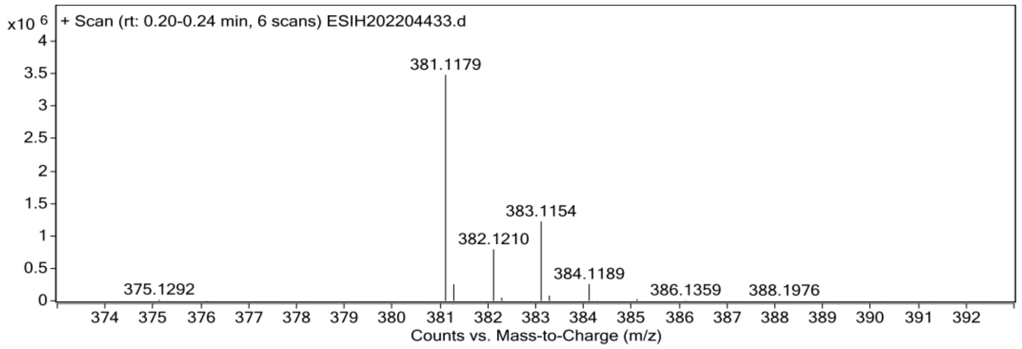

Formula Calculator Results

m/z	Calc m/z	Diff (mDa)	Diff (ppm)	Ion Formula	Ion
381.1179	381.1176	-0.28	-0.73	C19 H20 Cl F2 N2 O2	(M+H)+

Supplementary Fig. 16 HRMS spectrum of ZK11.

Data Filename	ESIH202204435.d	Sample Name	ZK24
Sample ID		Position	P1-B3
Instrument Name	Agilent G6520 Q-TOF	Acq Method	20160322_MS_ESIH_POS_1min.m
Acquired Time	11/3/2022 17:11:50	IRM Calibration Status	Success
DA Method	small molecular data analysis method.m	Comment	ESIH by fangsu

User Spectra

Fragmentor Voltage	Collision Energy	Ionization Mode
150	0	ESI

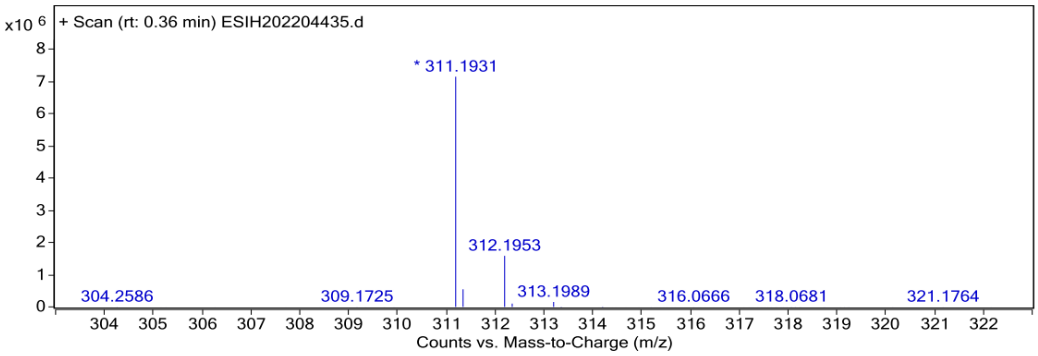

Formula Calculator Results

m/z	Calc m/z	Diff (mDa)	Diff (ppm)	Ion Formula	Ion
311.1931	311.1929	-0.11	-0.34	C17 H25 F2 N2 O	(M+H)+

Supplementary Fig. 17 HRMS spectrum of ZK24.

Data Filename	ESIH202204434.d	Sample Name	ZK53
Sample ID		Position	P1-B2
Instrument Name	Agilent G6520 Q-TOF	Acq Method	20160322_MS_ESIH_POS_1min.m
Acquired Time	11/3/2022 17:10:33	IRM Calibration Status	Success
DA Method	small molecular data analysis method.m	Comment	ESIH by fangsu

User Spectra

Fragmentor Voltage	Collision Energy	Ionization Mode
150	0	ESI

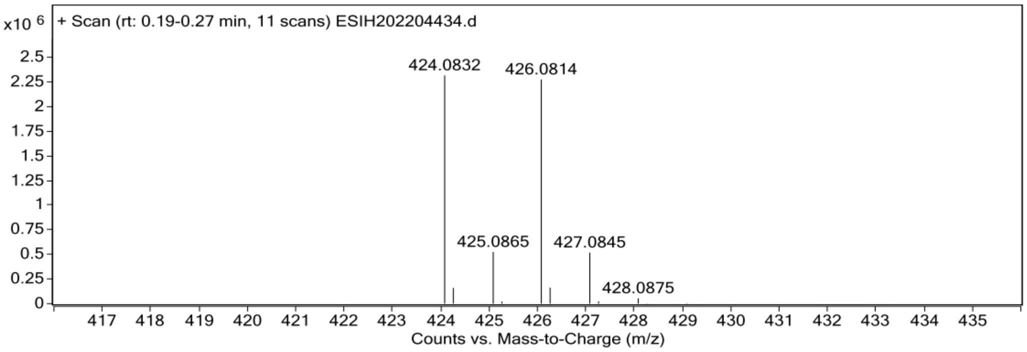

Formula Calculator Results

m/z	Calc m/z	Diff (mDa)	Diff (ppm)	Ion Formula	Ion
424.0832	424.0831	-0.13	-0.31	C19 H21 Br F2 N3 O	(M+H)+

Supplementary Fig. 18 HRMS spectrum of ZK53.
