# Supplementary material for: Selective activator of human ClpP triggers cell cycle arrest to inhibit lung squamous cell carcinoma
Source: Nat Commun. 2023 Nov 3;14:7069. doi: 10.1038/s41467-023-42784-4 (PMC10624687; doi:10.1038/s41467-023-42784-4)
Supplement: Supplementary file 3 — Reporting Summary [file 41467_2023_42784_MOESM3_ESM.pdf]

nature portfolio

Corresponding author(s): Cai-Guang Yang

Last updated by author(s): Sep 24, 2023

Reporting Summary

Nature Portfolio wishes to improve the reproducibility of the work that we publish. This form provides structure for consistency and transparency in reporting. For further information on Nature Portfolio policies, see our Editorial Policies and the Editorial Policy Checklist.

Statistics

For all statistical analyses, confirm that the following items are present in the figure legend, table legend, main text, or Methods section.

n/a	Confirmed	
☐	☒	The exact sample size (*n*) for each experimental group/condition, given as a discrete number and unit of measurement
☐	☒	A statement on whether measurements were taken from distinct samples or whether the same sample was measured repeatedly
☐	☒	The statistical test(s) used AND whether they are one- or two-sided<br>*Only common tests should be described solely by name; describe more complex techniques in the Methods section.*
☒	☐	A description of all covariates tested
☐	☒	A description of any assumptions or corrections, such as tests of normality and adjustment for multiple comparisons
☐	☒	A full description of the statistical parameters including central tendency (e.g. means) or other basic estimates (e.g. regression coefficient) AND variation (e.g. standard deviation) or associated estimates of uncertainty (e.g. confidence intervals)
☒	☐	For null hypothesis testing, the test statistic (e.g. *F*, *t*, *r*) with confidence intervals, effect sizes, degrees of freedom and *P* value noted<br>*Give P values as exact values whenever suitable.*
☒	☐	For Bayesian analysis, information on the choice of priors and Markov chain Monte Carlo settings
☒	☐	For hierarchical and complex designs, identification of the appropriate level for tests and full reporting of outcomes
☒	☐	Estimates of effect sizes (e.g. Cohen's *d*, Pearson's *r*), indicating how they were calculated

Our web collection on statistics for biologists contains articles on many of the points above.

Software and code

Policy information about availability of computer code

Data collection	Differential scanning fluorimetry data were collected using CFX manager 3.1<br>Nano differential scanning fluorimetry data were collected using NanoTemper, Prometheus NT.48<br>Flow cytometry data were collected using CellQuest Pro v6.0<br>Seahorse data were collected using Seahorse XF96 v1.4<br>Cytotoxicity assay data were collected by Tecon SPARKCONTROL v2.2<br>Diffraction data of ZK53/HsClpP were collected via Finback 1.0.

Data analysis	All statistical analysis were performed 1n Graph Pad Prism v8.3.0<br>Quantitative analysis was performed using ImageJ v1.51j8<br>Molecular alignments were performed in PyMOL v1.8.x<br>The sequence alignment was performed using ClustalW2 and ENDscript/ESPript 3<br>The models of HsClpP (PDB code 1TG6) were built in COOT 0.8.1<br>Diffraction data of ZK53/HsClpP was automatically processed by Aquarium and refined with the program refmac 5.0<br>Differential scanning fluorimetry data analysis were performed with Bio-Rad CFX Manager 3.1<br>Nano differential scanning fluorimetry data were performed using PR. ThermControl, v2.1.6<br>Cell cycle data were performed using ModFit LT v3.1<br>Flow cytometry data were performed using FlowJo vX.0.7<br>Seahorse data were performed using Wave 2.6.3<br>Comet assay data were performed using the plugin OpenComet v1.3

For manuscripts utilizing custom algorithms or software that are central to the research but not yet described in published literature, software must be made available to editors and reviewers. We strongly encourage code deposition in a community repository (e.g. GitHub). See the Nature Portfolio guidelines for submitting code & software for further information.

Data

Policy information about availability of data

All manuscripts must include a data availability statement. This statement should provide the following information, where applicable:

- Accession codes, unique identifiers, or web links for publicly available datasets
- A description of any restrictions on data availability
- For clinical datasets or third party data, please ensure that the statement adheres to our policy

The atomic coordinates and structure factors data generated in this study have been deposited in the Protein Data Bank (PDB, www.pdb.org) under accession code 8HGK [http://doi.org/10.2210/pdb8HGK/pdb] for ZK53/HsClpP. Other X-ray structural data used in this study are available in the PDB database under accession code 1TG6 [http://doi.org/10.2210/pdb1TG6/pdb]. The amino acid sequence can be found at the National Center for Biotechnology Information (NCBI, https://www.ncbi.nlm.nih.gov/) with the accession number NP_006003 [https://www.ncbi.nlm.nih.gov/protein/NP_006003.1] for HsClpP; KFL07692 [https://www.ncbi.nlm.nih.gov/protein/KFL07692.1] for SaClpP. The processed TCGA data are available under restricted access for copyright, access can be obtained by UALCAN (https://ualcan.path.uab.edu/index.html) created by the university of Alabama at Birmingham. The RNA-seq data generated in this study have been deposited in NCBI SRA dataset under accession code PRJNA902171 [https://www.ncbi.nlm.nih.gov/sra/PRJNA902171]. Source data are provided with this paper.

Research involving human participants, their data, or biological material

Policy information about studies with human participants or human data. See also policy information about sex, gender (identity/presentation), and sexual orientation and race, ethnicity and racism.

Reporting on sex and gender	N/A
Reporting on race, ethnicity, or other socially relevant groupings	N/A
Population characteristics	N/A
Recruitment	N/A
Ethics oversight	N/A

Note that full information on the approval of the study protocol must also be provided in the manuscript.

Field-specific reporting

Please select the one below that is the best fit for your research. If you are not sure, read the appropriate sections before making your selection.

[x] Life sciences [ ] Behavioural & social sciences [ ] Ecological, evolutionary & environmental sciences

For a reference copy of the document with all sections, see nature.com/documents/nr-reporting-summary-flat.pdf

Life sciences study design

All studies must disclose on these points even when the disclosure is negative.

Sample size	No statistical methods were used to determine sample sizes. Sample sizes were determined on the basis of previous experimental experience (Nat Commun. 2022, 13, 6909;Cell Chem. Biol. 2022, 29, 1396). Sample sizes were sufficient to perform statistical analyses.
Data exclusions	No samples were excluded.
Replication	The number of independent experiments/mice/samples are mentioned in the figure legends. All attempts at replication are successful. All blots and gels were performed in triplicate and a single experimental image is shown. xenograft and autochthonous mouse models, H&E

	staining and immunohistochemistry were performed once. Three representative images of H&E staining and immunohistochemistry were taken in each sample and one is shown. All images recorded on H&E staining indicated a similar trend.
Randomization	All mice were randomly allocated into different groups. Other samples, such as biochemical, cell, or microorganism samples, were maintained or cultured in the same environment and randomly allocated into different groups.
Blinding	For the in vivo experiments, investigators who were designing the organization of the groups and performing the treatment were not blinded to the groups and treatment, while the investigators collecting data such as tumor volume and tumor weight were blinded. The investigators were blinded to group allocation during data analysis. The investigators did not know the group information of each sample. Other in vitro experiments were not performed blindly because these results can be directly and easily obtained from instrument measurements or visual observations.

Reporting for specific materials, systems and methods

We require information from authors about some types of materials, experimental systems and methods used in many studies. Here, indicate whether each material, system or method listed is relevant to your study. If you are not sure if a list item applies to your research, read the appropriate section before selecting a response.

Materials & experimental systems

n/a	Involved in the study
☐	☒ Antibodies
☐	☒ Eukaryotic cell lines
☒	☐ Palaeontology and archaeology
☐	☒ Animals and other organisms
☒	☐ Clinical data
☒	☐ Dual use research of concern
☒	☐ Plants

Methods

n/a	Involved in the study
☒	☐ ChIP-seq
☐	☒ Flow cytometry
☒	☐ MRI-based neuroimaging

Antibodies

Antibodies used	Antibodies of HsClpP (1:2,000, Clo#EPR7133, Cat#ab124822, Lot#GR3210822-7, Abcam), NDUFB8 (1:3,000, Clo#EPR15961, Cat#ab192878, Lot#GR243097-1, Abcam), NDUFA12 (1:2,000, Clo#EPR15867-28, Cat#ab192617, Lot#GR3209820-3, Abcam), SDHA (1:3,000, Clo#5E10G12, Cat#66588-1-Ig, Lot#10006838, Proteintech), SDHB (1:10,000, Clo#EPR10880, Cat#ab175225, Lot#GR3380267-7, Abcam), UQCRC2 (1:2,000, Clo#EPR13051, Cat#ab203832, Lot#GR247396-11, Abcam), COX IV (1:1,000, Cat#ab153709, Lot#GR291729-10, Abcam), p-Rb S795 (1:1,000, Cat#AP0088, Lot#2100940201, Abclonal), p-Rb T826 (1:1,000, Clo#EPR5351, Cat#ab133446, Lot#GR96233-11, Abcam), p-Rb S807/811 (1:1,000, Clo#D20B12, Cat#8516T, Lot#9, CST), Cyclin D1 (1:10000, Clo#EPR2241, Cat#ab134175, Lot#GR3212345-11, Abcam), Cyclin E2 (1:1,000, Clo#ARC1515, Cat#A9305, Lot#4000001515, Abclonal), CDK2 (1:1,000, Cat#A0294, Lot#0600070401, Abclonal), PCNA (1:1,000, Clo# ARC51325, Cat#A12427, Lot#4000002488, Abclonal), γ-H2AX (1:1,000, Clo#20E3, Cat#9718S, Lot#13, CST), p-ATM S1981 (1:1,000, Clo#EP1890Y, Cat#ab81292, Lot#GR3285525-7, Abcam), p-CHK2 T68 (1:1,000, Clo#C13C1, Cat#2197T, Lot#12, CST), p-CHK1 S345 (1:1,000, Clo#133D3, Cat#2348T, Lot#18, CST) CDC25A (1:500, Clo#P30304, Cat#sc-7389, Lot#D2821, Santa Cruz), p-ATR T1989 (1:1,000, Cat#GTX128145, Lot#44384, GeneTex), Ki67 (1:200, Clo#SP6, Cat#ab16667, Lot#GR10004156, Abcam), GAPDH (1:5,000, Clo#1E6D9, Cat#60004-1-Ig, Lot#10025237, Proteintech), β-actin (1:5,000, Clo#2D4H5, Cat#66009-1-Ig, Lot#10004156, Proteintech), SOX2 (1:1,000, Clo#EPR3131, Cat#ab92494, Lot#GR3285529-6, Abcam), Cytokeratin 5 (KRT5) (1:1,000, Clo#XS20200904015, Cat#BS1208, Lot#XCJ36131, Bioworld), p40 (1:200, Clo#ZR8, Cat#RMA-0815, Lot#2108120815C5, MXB Biotechnologies), TTF1 (1:500, Clo#EPR8190-6, Cat#ab133638, Lot#GR3431564-1, Abcam), HRP-conjugated goat anti-rabbit IgG (1:10,000, Cat#CW0103, Cwbio), and HRP-conjugated goat anti-mouse IgG (1:10,000, Cat#CW0102, Cwbio) were commercially purchased. Antibodies of SaClpP (1:5,000, Cat#C11185) and SaGAPDH (1:5,000, Cat#C1399) were generated by Shanghai Immune Biotech Co., Ltd. using the purified proteins as the antigen and validated by ELISA experiments.
Validation	SaClpP and SaGAPDH antibodies were validated by the manufacturer using ELISA experiments. The validation reports are provided by the manufacturer and are available on request. All of the two antibodies have been utilized for immunoblot in the previous publications from our lab (Nat. Commun. 2022, 13, 6909)<br>HsClpP (https://www.abcam.cn/products/primary-antibodies/clpp-antibody-epr7133-ab124822.html); NDUFB8 (https://www.abcam.cn/products/primary-antibodies/ndufb8-antibody-epr15961-ab192878.html), NDUFA12 (https://www.abcam.cn/products/primary-antibodies/dap13ndufa12-antibody-epr15867-28-ab192617.html), SDHB (https://www.abcam.cn/products/primary-antibodies/sdhb-antibody-epr10880-ab175225.html), UQCRC2 (https://www.abcam.cn/products/primary-antibodies/uqcrc2-antibody-epr13051-ab203832.html), COX IV (https://www.abcam.cn/products/primary-antibodies/cox-iv-antibody-mitochondrial-marker-ab153709.html), p-Rb S795 (https://abclonal.com.cn/catalog/AP0088), p-Rb T826 (https://www.abcam.cn/products/primary-antibodies/rb-phospho-t826-antibody-epr5351-ab133446.html), p-Rb S807/811 (https://www.cellsignal.cn/products/primary-antibodies/phospho-rb-ser807-811-d20b12-xp-rabbit-mab/8516?site-search-type=Products&N=4294956287&Ntt=8516t&fromPage=plp&_requestid=3471534), Cyclin D1 (https://www.abcam.cn/products/primary-antibodies/cyclin-d1-antibody-epr2241-c-terminal-ab134175.html), Cyclin E2 (https://abclonal.com.cn/catalog/A9305), CDK2 (https://abclonal.com.cn/catalog/A0294), PCNA (https://abclonal.com.cn/catalog/A12427), γ-H2AX (https://www.cellsignal.cn/products/primary-antibodies/phospho-histone-h2a-x-ser139-20e3-rabbit-mab/9718?site-search-type=Products&N=4294956287&Ntt=9718s&fromPage=plp&_requestid=3471649), p-ATM S1981 (https://www.abcam.cn/products/primary-antibodies/atm-phospho-s1981-antibody-ep1890y-ab81292.html), p-CHK2 T68 (https://www.cellsignal.cn/products/primary-antibodies/phospho-chk2-thr68-c13c1-rabbit-mab/2197?site-search-type=Products&N=4294956287&Ntt=2197t&fromPage=plp&_requestid=3471757), p-CHK1 S345 (https://www.cellsignal.cn/

products/primary-antibodies/phospho-chk1-ser345-133d3-rabbit-mab/2348?site-search-type=Products&N=4294956287&Ntt=2348t&fromPage=plp&_requestid=3471810) CDC25A (https://www.scbt.com/zh/p/cdc25a-antibody-f-6), p-ATR T1989 (https://www.genetex.cn/Product/Detail/ATR-phospho-Thr1989-antibody/GTX128145), Ki67 (https://www.abcam.cn/products/primary-antibodies/ki67-antibody-sp6-ab16667.html), GAPDH (https://www.ptgcn.com/products/GAPDH-Antibody-60004-1-Ig.htm), β-actin (https://www.ptgcn.com/products/Pan-Actin-Antibody-66009-1-Ig.htm), SOX2 (https://www.abcam.cn/products/primary-antibodies/sox2-antibody-epr3131-ab92494.html), Cytokeratin 5 (KRT5) (https://www.antibodypedia.com/gene/3501/KRT5/antibody/1560432/BS1208. The website did not contain validation, but this antibody are widely used in the literature, e.g., doi: 10.1016/j.ccell.2015.04.001.), p40 (http://www.maxim.com.cn/sitecn/dklkthdklkt/7500.html#. The website did not contain validation, but this antibody are widely used in the literature, e.g., doi: 10.1177/10732748221087075), TTF1 (https://www.abcam.cn/products/primary-antibodies/ttf1-antibody-epr8190-6-ab133638.html),

Eukaryotic cell lines

Policy information about cell lines and Sex and Gender in Research

Cell line source(s)

H1703 (ATCC, human-derived, male)
H520 (ATCC, human-derived, male)
H226 (Cell bank of the Chinese Academy of Sciences, Shanghai, China, human-derived, male)
SK-MES-1 (Cell bank of the Chinese Academy of Sciences, Shanghai, China, human-derived, male)
MRC-5 (Cell bank of the Chinese Academy of Sciences, Shanghai, China, human-derived, male)
HEK293T/17 (Cell bank of the Chinese Academy of Sciences, Shanghai, China, isolated from human embryo kidney tissue)

Authentication

The cells were not authenticated using STR.

Mycoplasma contamination

The cell lines were tested negative for mycoplasma contamination.

Commonly misidentified lines
(See ICLAC register)

No commonly misidentified line was used in this study.

Animals and other research organisms

Policy information about studies involving animals; ARRIVE guidelines recommended for reporting animal research, and Sex and Gender in Research

Laboratory animals

Six-week-old female Balb/c mice were purchased from Shanghai Jihui Laboratory Animal Care Co., Ltd. The KrasLSL-G12D/+;Lkb1fl/fl mice at 6-8 weeks were from Hongbin Ji lab (Center for Excellence in Molecular Cell Science, China). S. aureus Newman was from Lefu Lan lab (Shanghai Institute of Materia Medica, China). The gut microbioes were from Kan Ding lab (Shanghai Institute of Materia Medica, China).

Wild animals

The study did not involve wild animals.

Reporting on sex

Sex has not been confirmed as a critical factor in this study. Although there may be slight variations in tumor progression between males and females, for the sake of consistency in the experiments, the same sex was selected for the CDX and KL mouse models. Further investigations are required to determine gender differences.

Field-collected samples

No filed-collected samples was used in this study.

Ethics oversight

The xenograft mice models were performed in accordance with the Institutional Animal Care and Use Committee (IACUC) of the Shanghai Institute of Materia Medica. For the animal used in the genetically engineered mouse model, all animal procedures were performed under the ethical guidelines of the Center for Excellence in Molecular Cell Science, Chinese Academy of Sciences.

Note that full information on the approval of the study protocol must also be provided in the manuscript.

Flow Cytometry

Plots

Confirm that:

- [x] The axis labels state the marker and fluorochrome used (e.g. CD4-FITC).
- [x] The axis scales are clearly visible. Include numbers along axes only for bottom left plot of group (a 'group' is an analysis of identical markers).
- [x] All plots are contour plots with outliers or pseudocolor plots.
- [x] A numerical value for number of cells or percentage (with statistics) is provided.

Methodology

Sample preparation

Cell cycle analysis: The LUSC cells were seeded into 60-mm dishes and allowed to adhere for 24 h before adding ZK53. After 48 h, the samples were prepared for cell cycle analysis using the Cell Cycle and Apoptosis Analysis Kit (Meilunbio) according to the manufacturer's instructions. The samples were then analyzed by a BD FACSCalibur flow cytometer, and the cell cycle phase distribution was analyzed by ModFit LT software.

Apoptosis analysis: The LUSC cells were seeded into 6-well plates. After adhering for 24 h, 1 μM ZK53 was added, and the cells were treated at different times as indicated. Then the cells were harvested, washed with PBS, and stained with annexin V-FITC using the Annexin V-FITC/PI Apoptosis detection Kit (Meilunbio). The samples were analyzed by a BD FACSCalibur flow cytometer. The proportion of apoptotic cells (annexin V-positive) was analyzed using FlowJo software.

mtROS measurement: The LUSC cells were seeded into 6-well plates, and after adherence, the cells were treated with ZK53 at the indicated concentration. After treatment for 48 h, the cells were collected and washed three times with pre-warmed Hanks Balanced Salt Solution (HBSS, Meilunbio). Then the cells were treated with 5 μM MitoSOX (Invitrogen) in the dark at 37°C for 20 min and washed with HBSS. The fluorescence intensity was determined by BD FACSCalibur flow cytometer and analyzed using FlowJo software.

Mitochondrial membrane potential: The Enhanced Mitochondrial Membrane Potential Assay Kit with JC-1 (Beyotime) was used according to the manufacturer's instructions. Briefly, after treatment with ZK53 for 72 h, the LUSC cells were collected and resuspended in a JC-1 working solution. The cells were incubated for 20 min at 37°C in the dark, washed three times, and analyzed using the BD FACSCalibur flow cytometer. The relative MMP level was calculated using the red/green fluorescence intensity median and normalized to the DMSO-treated group.

Instrument	BD FACSCalibur™ Flow Cytometer
Software	Collection: CellQuest Pro v6.0; analysis: FlowJo vX.0.7
Cell population abundance	No sort was performed in the study.
Gating strategy	Initial cell populations were gated using FSC and SSC plots of unstained cell control sample to remove cell debris. Doublets and cell aggregates were excluded by gating in single cells (FL2-1 vs. FL2-W) in cell cycle analysis: The cell population gated in after debris and exclusion were then used to create single-staining histograms (cell cycle based on PI, apoptosis based on annexin V-FITC, mtROS based on MitoSOX).

☒ Tick this box to confirm that a figure exemplifying the gating strategy is provided in the Supplementary Information.
